# Knowledge, Attitude, Practice, and Adherence to Antiretroviral Therapy among People Living with HIV in Nepal

**DOI:** 10.1155/2023/7292115

**Published:** 2023-07-17

**Authors:** Sweta Shrestha, Subodh Chataut, Badri Kc, Khagendra Acharya, Sait Kumar Pradhan, Sunil Shrestha

**Affiliations:** ^1^Department of Pharmacy, School of Science, Kathmandu University, Dhulikhel, Kavre, Nepal; ^2^Department of Management Informatics and Communication, School of Management, Kathmandu University, Dhulikhel, Kavre, Nepal; ^3^Department of Clinical Physiology, Maharajgunj Medical Campus, Institute of Medicine, Kathmandu, Nepal; ^4^School of Pharmacy, Monash University Malaysia, Subang Jaya, Selangor, Malaysia

## Abstract

**Introduction:**

Patient's knowledge and attitude towards their treatment avert stereotypical misconceptions about the disease and its treatment, as well as aid in attaining optimal adherence. This study investigated the knowledge, attitude, practice, and adherence of antiretroviral therapy (ART) clients in Nepal.

**Method:**

A cross-sectional study was conducted among 165 ART clients visiting five ART sites in the far western region and the capital city of Nepal. The convenience sampling method was employed, and the data were collected through interviews with ART clients using a validated questionnaire. Binary logistic regression was used to identify associated factors.

**Result:**

Approximately 80.7% had adequate knowledge and 55% had a positive attitude towards ART. Stigmatization was associated with ARV by only 4.2%. Half of the participants (50.3%) revealed that they had surreptitiously stored their ART medication, diverging from the recommended storage guidelines. A significant proportion of respondents (33.3%) chose to repackage the medication as a strategy to prevent unintended disclosure of their HIV status. Many (59.3%) believed that ART does not prolong life. Nevertheless, they advocated the regular use of ART rather than taking it only when the health deteriorates (81.8%). The majority (97.6%) were found to be adherent to their ART. There was a significant association of age with a level of knowledge and attitude (*p* < 0.05). A significant association was also found between knowledge and attitude towards ART (*p* < 0.05). None of the variables had a significant association with adherence (*p* > 0.05).

**Conclusion:**

Overall, adequate knowledge was demonstrated, whereas efforts are still needed to improve the attitude of ART clients towards ART. A need for counseling regarding the storage practices of ART is needed. A focus on ensuring the perfect translation of adequate knowledge and a positive attitude to the practice of ART clients is essential. Whether adequate ART knowledge and attitude scores will lead to near-perfect ART adherence needs further investigation.

## 1. Introduction

Human Immunodeficiency Virus (HIV) infection and Acquired Immuno Deficiency Syndrome (AIDS) have emerged as a formidable global public health crisis claiming 36.3 million lives as of year 2021 [1]. Nepal, one of the countries in South Asia, has recently confronted a meteoric inclination in HIV infection rates, with the recently reported number of people living with HIV (PLHIV) being 29,503. Antiretroviral therapy (ART) was marked as a revolutionary triumph against HIV/AIDS [2]. An antiretroviral therapy service was initiated in Nepal in 2004 by Sukraraj Tropical and Infectious Disease Hospital. To universalize access to ART, the Nepal Government has made ART freely available for all PLHIV, along with the nationwide expansion of 80 ART sites and 20 ART dispensing centers (ADCs), covering 61 districts in Nepal. Community and home-based care (CHBC) services have been initiated to provide essential services such as prevention, counseling, nutritional support, and education on treatment for improving adherence [3]. Adherence above 95% is the benchmark for a successful ART program which otherwise might lead to a sequel of increased HIV viral load, fueling HIV transmission and the emergence of resistant strains [4–9]. Hence, curbing viral replication and achieving optimal clinical benefits contribute to a near-perfect adherence to ART.

Patients' knowledge and attitudes towards treatment play crucial roles in adherence to ART. Understanding the medication regimen and the disease itself empowers individuals to address misconceptions and overcome barriers to adherence [10, 11]. Understanding one's medication regimen and disease tends to give the individual cues to identify and revoke misconceptions about the disease and its treatment which are the principal threats to adherence [12–15]. Improving knowledge of disease and treatment regimens as well as the patient's attitude towards the regimen should be targeted to address the inconsistencies of adherence [16]. Despite the importance of knowledge and attitudes in ART adherence, limited study has been conducted in Nepal specifically addressing the knowledge, attitude, and practice of PLHIV towards HIV/AIDS only [2]. This research gap underscores the need to explore and understand the unique challenges faced by PLHIV in Nepal, shedding light on their knowledge levels, attitudes towards ART, and adherence practices.

In line with this rationale, this study aimed to assess the knowledge, attitude, practice, and adherence of PLHIV towards ART in Nepal. By examining these key factors, we aim to identify areas for improvement and develop targeted interventions to enhance adherence and optimize treatment outcomes.

## 2. Materials and Methods

### 2.1. Study Design, Study Site, and Population Characteristics

Multicentric cross-sectional study was conducted at five ART centers: Teku Hospital, Kathmandu; Bhaktapur Hospital, Bhaktapur; Mahakali Hospital, Mahendranagar; Dadeldhura Hospital; and Doti District Hospital. All the patients visiting the ART were receiving the ART during the study period.

The study population consisted of PLHIV who visited the selected ART sites during the study period of 6 months from August 9, 2021 to February 10, 2022. ART clients, who were 18 years old, had been on the ART for at least three months, and agreed to provide written consent to participate in the study were included in the study. ART clients with the coexisting condition of pregnancy, pediatrics, and the patients admitted to the hospital were excluded.

### 2.2. Sampling Method and Technique

Convenience sampling technique was used for selection of the participants who met the inclusion criteria.

The sample size for this cross-sectional study was calculated using the following formula:

Sample size for adherence [*p*=0.874] [17]

(1) $$\begin{array}{c} n=z^{2}pqN/\left[\left(N-1\right)e^{2}+z^{2}.p.q\right]\\ =1.96^{2}*0.874*\left(1-0.874\right)*2947/\left[\left(2947-1\right)0.05^{2}+1.96^{2}\times 0.874\times 0.126\right]\\ =160\text{.} \end{array}$$

Sample size for KAP [*p*=0.876] [18]

(2) $$\begin{array}{c} n=z^{2}pqN/\left[\left(N-1\right)e^{2}+z^{2}.p.q\right]\\ =1.96^{2}*0.876*\left(1-0.876\right)*2947/\left[\left(2947-1\right)0.05^{2}+1.96^{2}\times 0.876\times 0.124\right]\\ =158\text{.} \end{array}$$

Table 1 shows the sample size stratification in different districts/hospitals.

### 2.3. Variables

#### 2.3.1. Independent Variables

The independent variables included sociodemographic variables including age, gender, religion, education status, marital status, occupation, employment status, smoking habit, drug use habit, time to reach the ART site, disclosure of HIV status, infected duration, treatment duration, ART regimen, and educational intervention.

#### 2.3.2. Outcome Variables

The dependent variables included the knowledge, attitude, and practice (KAP) of PLHIV on ART and their adherence level.

### 2.4. Data Collection Tool

An interviewer-administered questionnaire (Supplementary File (available here)) was designed after an extensive review of the previous similar survey studies, and some modifications were made after consultation with experts [18, 19]. The questionnaire contains 5 parts that include the subjects' demographic details, their KAP of ART, and their adherence. The knowledge section includes 11 multiple-choice questions on ART. The attitude section has 8 Likert-like questions with strongly agree, agree, undecided, disagree, and strongly disagree choices. The score for each question ranges from +2 to −2 (+2, +1, 0, −1, and −2) for the correct statements. For incorrect statements, reverse scoring was done where the score ranged from −2 to +2 (−2, −1, 0, +1, and +2). Practice section contains 7 questions related to the users' practice of ART. Each correct answer scored 1 point, whereas the incorrect answer scored 0 points. For question number 1, the respondents scored 1 point only if they could name all three components of the fixed-dose regimen. Similarly, for question number 6, the respondents achieved 1 point only if they could mention suppressing the power of HIV and not curing the disease as the primary purpose of ART. Anyone selecting only one of the the correct options was given a 0.5 score. The total scores were 0–11 for knowledge and between −16 and +16 for attitude. Knowledge and attitude scores were converted to a percentage by dividing the total score of each respondent in each section by the maximum score of the same section. The practice of the interviewees was expressed in terms of frequency and percentage. The respondents scoring ≤50% were said to have inadequate knowledge, and those scoring >50% were said to have adequate knowledge on ART [19]. Similarly, the respondents scoring ≤50% were said to have a negative attitude, and those scoring >50% were said to have a positive attitude [19]. The development of the data collection tool was followed by validation of the tool. For content validation, the content validity ratio (CVR = (*ne* − *N*/2)/(*N*/2)), relevance content validity index (R-CVI), clarity content validity index (C-CVI), and simplicity content validity index (S-CVI) were calculated. Eight experts were asked to score the questionnaire items for necessity, relevance, clarity, and simplicity. As per Lawshe's Table, the significance level was considered 0.75. All the items had a CVR ≥0.75 and a CVI average was 0.96. None of the items required any modifications. Cronbach alpha value was 0.72, indicating good reliability. This was followed by translation validation, where the questionnaire was initially translated into Nepali language and then back-translated into English. The final version was compared with the original version of the questionnaire. The final version of the questionnaire was tested among 18 PLHIV in Mahakali Hospital.

Adherence was assessed based on patients' self-report, one of the simplest and most extensively used methods. It was calculated using the formula from the National HIV Testing and Treatment Guidelines, 2017. Adherence percentage = [number of pills taken during the specific period (1 month)/number of pills to be taken during that particular period (1 month)] × 100. Patients were categorized as ART adherent if adherence was ≥95% and nonadherent if adherence was <95% [8, 20].

### 2.5. Data Collection Process

PLHIV in Bagmati Province (Teku Hospital, Kathmandu and Bhaktapur Hospital, Bhaktapur) and Far West Province (Mahakali Hospital, Dadeldhura Hospital, and Doti District Hospital) were approached by the researcher, and the objectives of the study were explained. Written informed consent was obtained from each respondent, and assurance was conveyed regarding their voluntary participation and maintenance of their confidentiality. The interviewer administered the questionnaire to each PLHIV who agreed to participate in the study. All the PLHIV visiting the ART sites were receiving ART.

### 2.6. Statistical Analysis

The collected data were entered and analyzed using Statistical Package for Social Sciences (SPSS) version 26 (SPSS Inc., Chicago, IL, USA). Descriptive analyses were performed using frequencies and percentages. Pearson chi-square test (*χ*^2^) for independence was used to determine the association of adherence with patients' knowledge and attitude towards ART and to determine the association of patients' attitude towards ART with their corresponding knowledge level. A binary logistic regression analysis was done to identify determinant variables associated with knowledge, attitudes, and adherence. A *p* value <0.05 was considered statistically significant.

## 3. Results

### 3.1. Sociodemographic Characteristics

Majority (47.3%) were of the age group 39–58 years, male (58.8%), and Hindu (84.8%). Most were married (67.3%) and could only read and write without educational degrees (44.8%). The majority reported having never smoked (67.3%) or consumed alcohol (74.5%) in the past month (Table 2).

### 3.2. Knowledge of ART among PLHIV

Approximately 80.7% had adequate knowledge, while 19.3% had inadequate knowledge of ART. None of the respondents could name their ART. Most of them reportedly took their ART at a fixed time (99.4%) and were aware that ART should be taken for a lifetime (84.2%). Approximately 26.7% correctly stated that ART suppresses the power of HIV but does not cure the disease. The majority (58.2%) believed that ART decreases HIV viral load and increases CD4 cell count (53.9%), and missing doses reduces treatment efficiency (53.3%). Most (67.3%) opined that ART could successfully prevent mother-to-child HIV transmission (MTCT) and seemed to have realized the importance of ART adherence (77.6%) (Table 3).

### 3.3. Attitude of PLHIV to ART

Approximately 54.5% had a positive attitude towards ART. The majority believed that ART is currently the most effective available therapy to combat HIV (99.3%) and were convinced that they have HIV and need to be concerned about taking ART (95.1%). The majority (90.9%) was assured of the positive effect of ART on health and was persuaded by the fact that ART does more benefits than harm (79.3%). Very few (5.4%) felt that taking ART for one's lifetime is tiring and felt stigmatized by being on ARV therapy (4.2%). A maximal percentage (59.3%) presumed that ARV drugs have no role in prolonging the life of PLHIV and seemed to favor the regular use of ART (81.8%) (Table 4).

### 3.4. Practice of PLHIV towards Use of ART

A slight majority (50.3%) reportedly stored their ART “hidden and out of sight.” Most participants (*n* = 110, 66.7%) chose to store ART by transferring it to other plastic packaging, contrary to its storage in its original container (*n* = 55, 33.3%).

When asked about the action to be taken in case of missed doses, (*n* = 34, 20.6%) rightly mentioned taking the missed doses only if it was not near the time of the next dose, whereas (*n* = 124, 75.2%) opted for skipping the dose of ART medication that was missed. In the case of running out of prescription for ARV medication, (*n* = 132, 80%) defined their practice of borrowing from friends, whereas only one patient preferred to call and ask for a refill. Concerning the source of information about ART, (*n* = 145, 87.9%) seemed to have received information on ART from the ART dispensing staff, followed by referring physicians (*n* = 107, 64.8%). None of the patients had ever increased or decreased the dose of their ART on their own. Out of 165 participants, a considerable majority (*n* = 151, 91.5%) practiced self-medications, including the intake of antibiotics (*n* = 27, 16.4%), and very few reported taking herbal and ayurvedic drugs (*n* = 4, 2.4%) (Table 5).

### 3.5. Association of Demographic Characteristics and ART Knowledge and Attitude

Binary logistic regression was used to determine the association between sociodemographic characteristics and ART knowledge (Table 6). There was a significant association of age with a level of knowledge and attitude (*p* < 0.05). PLHIV in the age group 18–38 years were found to be 5.5 times more knowledgeable than those in the age group 38–58 years concerning ART (11.63 vs. 2.10). PLHIV in the age group 18–38 years were found to have 1.2 times more positive attitude than people in the age group 38–58 years (3.29 vs. 2.66). The other sociodemographic variables were not significantly associated with ART knowledge and attitude towards ART (*p* > 0.05) (Tables 6 and 7).

A significant association was found between ART knowledge and attitude (*p* < 0.05) (Table 8).

### 3.6. Adherence of PLHIV to ART

Most participants (97.6%) were found to be adherent to their ART. Reportedly, (*n* = 159, 6.4%) had never missed a dose of their ART drugs. When asked about the probable reasons for missing the ART doses by the ART users, being too busy at work (*n* = 102, 61.8%), staying away from home (*n* = 85, 51.5%), forgetting to take medicine (*n* = 62, 37.6%), feeling better and hence not taking the pills (*n* = 58, 35.2%), and feeling depressed (*n* = 51, 30.9%) were some of the major reasons stated by the respondents. A single respondent gave multiple answers to this question. None of the respondents used reminder devices to take their ART, whereas (*n* = 46, 27.9%) sought help from their family members to remember their ART doses (Table 9).

### 3.7. Patients' Adherence to Antiretroviral Medicines with Various Predictor Variables

Patients' adherence to antiretroviral medicines was not significantly related to any of the model variables (Table 10).

## 4. Discussion

The present study reported an adequate level of knowledge on ART among most of the participating PLHIV; this finding is in congruence to the previous findings [18, 19, 21–24]. Still, it contradicted a report where a sizeable proportion of PLHIV in Brazil was unaware of the crucial information on the prescribed ART [7]. All the respondents were aware of their once-daily regimen to be taken at a fixed time. However, none were able to name their ART or mention the components of the combination, which agrees with the earlier reports where only 5.8% and 13.7% could correctly quote the brand name or the ART regimen constituent [18, 25]. Nevertheless, this contradicts the findings of Potchoo et al., where 44.4% could ideally name the ARVs in the fixed-dose regimen [23]. A misconception was observed among the majority of the PLHIV who were ignorant of the flexibility of the dolutegravir- (DTG-) based regimen regarding food intake.

All the ART clients in this study had been kept on the first-line ART regimen that consisted of 2 NRTIs (nucleoside reverse transcriptase inhibitors) and an INSTI (integrase strand transfer inhibitor), namely, TDF + 3TC + DTG (tenofovir, lamivudine, and dolutegravir; TLD). All the respondents perfectly knew their dosage regimen, including the number of tablets to be taken per dose, the frequency of daily intakes, and the times of drug intake. Contrary to the present study, most ART clients are reported to be using a treatment regimen comprising 2 NRTIs plus 1 non-nucleoside reverse transcriptase inhibitor (NNRTI), as reported by Yao et al. As of today, a DTG-based regimen where DTG is combined with 2 NRTIs, TLD is the preferred first-line regimen for PLHIV in Nepal. This switchover from the efavirenz-based regimen to the new regimen is backed by the findings of the HIV Drug Resistance study conducted by the Ministry of Health and Population (MoHP) that has identified the emergence of resistant strains to the earlier used ARV regimen. This transition, as recommended by the new National HIV Testing and Treatment Guidelines, 2020, is also primarily based on the WHO guidelines published in 2018. The guideline iterates that a DTG-based regimen in combination with two NRTIs results in greater and faster viral suppression, a lesser incidence of adverse effects, a higher retention rate on treatment, fewer drug interactions, and a lower risk of developing resistance to ART as compared to efavirenz- (EFV-) based regimens [26, 27].

A patient's medication knowledge has been defined as “the awareness of the drug name, purpose, administration schedule, adverse effects or side effects, or special administration instructions,” and knowledge about medications is one of the most prominent patient-related factors affecting their drug-taking behavior, especially in chronic diseases [28]. The majority of the PLHIV in the present study were unaware of the names of their ART regimen, which reflects the gap in the information provided by the physicians, pharmacists, or ART counselors [29]. Some respondents were ignorant of the duration for which ART should be taken. A few also believed that ART needs to be taken only for some years, in consistency with other literature [18, 30]. However, our result mirrors a percentage lower than that of Almeida and Vieira [7]. Regular interaction is required between healthcare professionals and PLHIV, where they must be reminded that HIV/AIDS is an incurable, chronic disease that demands strict adherence and persistent treatment [25]. The majority believed that ART decreases the viral load, increases the CD4 count, and acts by suppressing the power of HIV, but does not cure the disease.

Nevertheless, a noticeable percentage remained unaware of the facts mentioned above. This echoes the findings of the antecedent studies [18, 22, 25, 31] and reflects a reportedly lower percentage than presented by Almeida and Vieira [7]. The users must obviate ART's unrealistic benefits, such as curing an incurable disease that otherwise might negatively impact their treatment adherence [32]. Most respondents opined that ART has a role in preventing MTCT. In contrast, nearly one-third lacked comprehension in this regard, a finding in alignment with Raberahona et al. (61.5%) and Nachega et al. (89.5%) [18, 33]. MTCT has been stated as one of the most prevalent routes of transmission of HIV infection among children [34]. UNAIDS has spearheaded a comprehensive and accelerated approach towards eliminating MTCT by implementing various interventions. As WHO guidelines recommend, one such intervention is initiating and maintaining the HIV-infected pregnant or breastfeeding women on ART [35]. Lack of information on ART's effectiveness in preventing vertical transmission, especially among HIV-positive women, might undermine the efforts to end MTCT globally. The majority were aware of the 95–100% adherence requirement for ART efficiency, whereas a small percentage remained ignorant on this which is in line with previous literature [22]. Nearly half the respondents perceived that treatment efficiency can be maintained despite missing ART doses, a percentage higher than that reported by preceding studies [18, 22, 30, 33]. It is incumbent on us to bridge such knowledge gaps, as interruptions in treatment can fuel the emergence of ARV resistance [2].

The majority had a positive attitude towards the use of ART, which corroborates the findings of Raberahona et al. Almost all the respondents denied the existence of therapies more effective than ART to battle HIV (99.3%), contradicting what is reported in Madagascar and India, where a handful of respondents were not completely persuaded of the effectiveness of ART. As mentioned earlier, some of the study respondents opined that traditional healers are more reliant on ART for the treatment of HIV [18, 21]. In contrast, such a misconception was not expressed in our study. Delayed access to HIV testing and ART initiation among PLHIV has been documented as a sequel of medical pluralism resulting from diversion to traditional healing, causing a serious impediment to the HIV care cascade [36]. In low-resource settings like Nepal, where traditional healing receives high patronage, initiatives in education are an absolute necessity to avert such deflections of PLHIV. Paradoxically, some opined that ART does not prolong life; this figure is higher than the studies conducted in Nigeria [19, 22, 32]. The majority were convinced of being infected with HIV and agreed that ART does more benefit than the harm which translated into their favoring attitude towards the positive effect of ART on health. This is in line with other studies [18, 22]. Most did not perceive taking ARV drugs for a lifetime as exhausting and were against the idea that ART should be taken only when sick. This outcome may be accounted to the various support programs that the government was running at the time of the study that provided care and support to the PLHIV, facilitated them in viral load sampling, and helped the client with their medication counseling and usage of ART. A minority were ashamed to be on ARV therapy. The stigma and discrimination PLHIV confront is a considerable setback for disclosing their HIV status and willingness to initiate or retain ART therapy. Sociocultural factors may have constituted a massive impediment that made them apprehensive about taking ART at home or at work [37]. Stigma and discrimination are the recognized barriers to testing, treating, and retaining patients in care and a bottleneck in achieving the 90-90-90 target by 2021. Acknowledging the criticality of such sociocultural issues can pose, Nepal's National HIV/AIDS Strategy 2016–2021 has undertaken the reduction of stigma and discrimination as one of its commitments [38].

Although the majority of respondents claimed to be open about their ART, this was not seen to have translated into practice, as nearly 50% of the users acknowledged keeping their ART hidden and out of sight. Only less than half of the study population followed the storage recommendation given by the manufacturers, and the majority reported the practice of self-repackaging. PLHIV are involved in self-repackaging of ART, where they transfer the medication from the original repository to other containers in an attempt to camouflage it out of an apprehension of unwanted disclosure of their HIV status and getting stigmatized. Such self-repackaging obviates the protection provided by the original packaging against external conditions such as light and moisture. Furthermore, patients fail to familiarize themselves with the essential drug-related instructions (expiration dates, maintenance of the fixed dosing time, and taking the drug concerning food intake) printed on the packaging [39].

The majority opted to skip the missed ART dose, one of the drivers for antiretroviral resistance [7]. Most were engaged in self-medication practices; some reported taking acid suppressants and herbal or ayurvedic medications concurrently with ART. The other reviews were divided mainly between antibiotics, antipyretics, and analgesics. Self-medication has been common among PLHIV, mainly due to the adverse effects of ARV, their willingness to get better, and for alleviating mild symptoms. Less aware are the patients that potential interactions with the self-medicated products may compromise the efficacy or lead to toxicity of ARVs. Around 50 to 80% of PLHIV did not confide in their self-medication practice to their physicians. This calls for integrating self-medication practice questions in medication history taking by physicians and pharmacists and ruling out the chances of potential interactions [39].

Most respondents were found to adhere to their ART regimen in congruence to previous studies in Nepal [8, 17]. A greater percentage of PLHIV has been found to adhere to their ART regimen compared to other studies [4, 23, 40, 41]. This reflects increased awareness of PLHIV on ART, possibly accounting for CHBC services initiated in Nepal and additional support programs [8]. Regarding probable reasons for missing ART doses, most respondents stated remaining too busy with other works, staying away from home, forgetting and feeling better, and hence, missing out on some of the doses. This is similar to previous findings [15, 22, 23]. The PLHIV might carry fewer reserves of their ART tablets and possibly miss the refill appointments due to being away from their homes and their working allegiance [42]. Some PLHIV patients may also overestimate the improvement in their health and miss some of the doses of ART. Hence, physicians or ART counselors should not overestimate their patients' adherence and should understand that continuous patient education and counseling are essential to ensure optimal adherence to ART and retention in treatment [19].

### 4.1. Strengths, Limitations, and Recommendations

This study represents a novel inquiry into the understanding, disposition, and behavior of people living with HIV (PLHIV) concerning their antiretroviral therapy (ART). Specifically, it includes participants from the far western regions of Nepal, which have been identified as high-risk areas for HIV transmission in accordance with the National HIV Strategic Plan 2016–2021, primarily due to heightened male labor migration to India. The constraints imposed by the limited timeframe of the study necessitated the utilization of a convenience sampling method. Subsequent stages of research can entail the development of educational interventions targeting specific population subsets, including pediatric patients and pregnant women with HIV.

## 5. Conclusion

An adequate knowledge of PLHIV on ART was observed. However, improving their attitude towards ART requires attention. Education to the concerned individuals should incorporate elements such as ART names, their mechanism of action, the harms of missed doses, and its' role in prolonging life. The findings of this study indicate that despite possessing good knowledge and a positive attitude towards ART, the majority of ART clients reported hiding their medication and not following the manufacturer's recommended storage guidelines to prevent the inadvertent disclosure of their HIV status. Although adherence to ART was high among the clients, it is necessary to conduct further investigations to determine if sufficient knowledge and positive attitudes towards ART can result in nearly perfect adherence.

## Figures and Tables

**Table 1:** Sample size stratification.

District/hospital	Total estimated HIV population	Calculation	Required number
Kathmandu/Teku Hospital	1996	(1996/2947) × 165 = 111.75	112
Bhaktapur/Bhaktapur Hospital	95	(95/2947) × 165 = 5.3	5
Doti/Doti Hospital	490	(490/2947) × 165 = 27	27
Dadeldhura/Dadeldhura Hospital	61	(61/2947) × 165 = 3.5	4
Kanchanpur/Mahakali Hospital	305	(305/2947) × 165 = 17	17
Total	2947		165

**Table 2:** Patient demographic characteristics (*n* = 165).

Variables	Frequency (%)
Age (in years)	
18–38	68 (41.2)
39–58	78 (47.3)
59–78	19 (11.5)
Sex	
Male	97 (58.8)
Female	68 (41.2)
Education level	
Can't read and write (i.e., illiterate)	43 (26.1)
Just read and write	74 (44.8)
Secondary	37 (22.4)
Bachelor	11 (6.7)
Occupation	
Service	2 (1.2)
Business	23 (13.9)
Labor	24 (14.5)
Agriculture	99 (60)
Unemployed	10 (6.1)
Other	7 (4.2)
Smoking habit in the past 1 month	
Never	111 (67.3)
1-2 cigarettes per day	40 (24.2)
3-4 cigarettes per day	7 (4.2)
More than four cigarettes per day	7 (4.2)
Alcohol consumption habit in the past 1 month	
Never	123 (74.5)
Once a month	23 (13.9)
2-3 times a month	15 (9.1)
2-3 times a week	2 (1.2)
3-4 times a week	1 (0.6)
Daily	1 (0.6)
Drug addiction history	
Never	154 (98.3)
In the past, but not currently	1 (0.6)
Yes, currently	10 (6.1)
HIV diagnosed	
Less than a year ago	1 (0.6)
1–3 years ago	6 (3.6)
More than 3 years ago	158 (95.8)
Start of ART	
Less than a year ago	4 (2.4)
1–3 years ago	6 (3.6)
More than 3 years ago	155 (93.9)
Disclosure of HIV status	
No	7 (4.2)
Yes	158 (95.8)
Educational intervention received	
No	6 (3.6)
Yes	159 (96.4)

**Table 3:** Knowledge of PLHIV towards antiretroviral therapy (*n* = 165).

Questions	Correct response frequency (%)
(1) What is the name of your ART medication?	
TDF + 3TC + DTG	0
ABC + 3TC + DTG	0
AZT + 3TC + DTG	0
AZT + 3TC + ATV/*r* (or LPV/*r*)	0
(2) How many tablets should you take each day for your ART medication?	
One tab	165 (100)
Two tab	0
Three tab	0
Four tab	0
Five tab	0
Six tab	0
(3) How should you take your ART?	
At a fixed time	164 (99.4)
At a variable time	1 (0.6)
(4) How do you take your ART in relation to food intake?	
After food	128 (77.6)
Half an hour before meal	1 (0.6)
Either with or without food	36 (21.8)
(5) How long should you take your ART?	
Lifelong	139 (84.2)
For some years	3 (1.8)
Can be stopped by doctor depending on CD4 count	0
Don't know	23 (13.9)
(6) What is the main purpose of ART?	
Suppress the power of HIV	56 (33.9)
It does not cure the disease forever	24 (14.5)
It suppress the power of HIV but does not cure the disease forever	44 (26.7)
Cure HIV/AIDS	0
Don't know	41 (24.8)
(7) What is the effect of ART on HIV viral load?	
Decreases HIV viral load	96 (58.2)
Increases HIV viral load	0
Does nothing to HIV viral load	1 (0.6)
Don't know	68 (41.2)
(8) What is the effect of ART on CD4 count?	
Decreases CD4 cells count	2 (1.2)
Increases CD4 cells count	89 (53.9)
Don't know	75 (45.5)
(9) What is the effect of ART on mother-to-child HIV transmission?	
It can prevent transmission	111 (67.3)
It can't prevent transmission	5 (3)
Don't know	50 (30.3)
(10) How much percentage of ART adherence is required to achieve optimum suppression of HIV viral load?	
Up to 94%	—
95–100%	128 (77.6)
Don't know	37 (22.4)
(11) Does missing your ART medication reduce treatment efficiency?	
Reduces treatment efficiency	88 (53.3)
Does not reduce treatment efficiency	77 (46.6)

**Table 4:** Attitude of PLHIV towards antiretroviral therapy (*n* = 165).

Variables	Frequency (%)				
	Strongly disagree	Disagree	Undecided	Agree	Strongly agree
(1) Believe to have effective therapy to treat HIV other than ART	81 (49.1)	83 (50.3)	1 (0.6)	—	-
(2) I needn't bother with ARV drugs since I'm not persuaded that I have HIV/AIDS	39 (23.6)	118 (71.5)	7 (4.2)	1 (0.6)	—
(3) ART has positive effect on health	—	4 (2.4)	11 (6.7)	122 (73.9)	28 (17)
(4) ART does more harm than benefit	17 (10.3)	114 (69.1)	29 (17.6)	4 (2.4)	1 (0.6)
(5) Taking ARV drugs for one's life time is tiring	83 (50.3)	64 (38.8)	9 (5.5)	8 (4.8)	1 (0.6)
(6) It is shameful to be on ARV therapy	107 (64.8)	47 (28.5)	4 (2.4)	5 (3)	2 (1.2)
(7) ARV drugs help to prolong life	28 (17)	70 (42.4)	36 (21.8)	25 (15.2)	6 (3.6)
(8) You should take ART only when you feel sick	36 (21.8)	99 (60)	27 (16.4)	3 (1.8)	-

**Table 5:** Practice of PLHIV towards antiretroviral therapy (*n* = 165).

Variables	Frequency (%)	
	Yes	No
(1) Where do you store your ART at home?		
Hidden and out of sight	83 (50.3)	81 (49.1)
Convenient storage but not necessarily as recommended by the dispenser	11 (6.7)	154 (93.3)
Storage that can help to remember daily schedule but not necessarily as recommend by dispenser	12 (7.3)	153 (92.7)
Suitable storage as recommended by the manufacturer	50 (30.3)	115 (69.7)
Storage out of the reach and sight of children	9 (5.5)	156 (94.5)
(2) How do you store your ART at home?		
By transferring to other plastic packaging	110 (66.7)	55 (33.3)
In its original carton or plastic packaging or bottle	55 (33.3)	110 (66.7)
(3) You just remembered that you forgot to take your evening ART medication dose yesterday. You would:		
Skip the dose of ART medication you missed	124 (75.2)	41 (24.8)
Take the missed ART medication dose right now only if it is not too close to the time of next dose	34 (20.6)	131 (79.4)
Wait and take 2 doses of ART medication this evening	—	165 (100)
Not missed	6 (3.6)	159 (96.4)
(4) If you ran out of your prescription for your ART medication you would:		
Borrow from friends	132 (80)	33 (20)
Call and ask for refills	1 (0.6)	164 (99.4)
Wait until your next appointment to get a new prescription	—	165 (100)
Others	39 (23.6)	126 (76.4)
(5) Where do you find out information about your ART?		
Referring physician	107 (64.8)	58 (35.2)
Internet	25 (15.2)	140 (84.8)
Patient information leaflet	20 (12.1)	145 (87.9)
ART dispensing staff	145 (87.9)	20 (12.1)
Other patients living with HIV	56 (33.9)	109 (66.1)
HIV/AIDS organizations	9 (5.5)	156 (94.5)
Others	2 (1.2)	163 (98.8)
(6) Have you ever increased or decreased the dose of your ART?	—	165 (100)
(7) Have you ever practiced self-medication?	151 (91.5)	14 (8.5)
If yes, what type of drugs have you already taken in self-medication?		
Antibiotics	47 (28.5)	118 (71.5)
Antipyretics	108 (65.5)	57 (34.5)
Drugs to relieve gastric acidity	27 (16.4)	138 (83.6)
Painkillers (analgesics)	69 (41.8)	96 (58.2)
Herbal and ayurvedic drugs	4 (2.4)	161 (97.6)
Others	2 (1.2)	163 (98.8)

**Table 6:** Binary logistic regression analysis of respondents' knowledge level on antiretroviral medicines with various predictor variables.

Predictors	*B* (SE)	*p* value	95% CI for OR [lower–upper]	Nagelkerke R2	Knowledge level	
					Adequate (*n*)	Inadequate (*n*)
Age (years)						
18–38	2.45 (0.69)	<0.001	11.63 [2.98–45.34]	0.158	64	4
39–58	0.74 (0.53)	0.161	2.10 [0.74–5.98]		58	20
Gender						
Male	0.28 (0.39)	0.469	1.33 [0.61–2.89]	0.005	79	18
Education level						
Illiterate	−1.67 (1.09)	0.126	0.18 [0.02–1.60]		28	15
Just read and write	−0.75 (1.09)	0.489	0.46 [0.05–3.99]	0.097	61	13
Secondary level	0.12 (1.20)	0.918	1.13 [0.10–12.12]		34	3
Occupation						
Service holders	0 (32226.05)	1.000	1.00 [0–]		2	0
Businessman	−19.64 (15191.49)	0.999	0	0.084	19	4
Labor	−18.80 (15191.49)	0.999	0		22	2
Agriculture	−20.11 (15191.49)	0.999	0		74	25
Unemployed	−19.00 (15191.49)	0.999	0		9	1
Smoking habit						
Never smoked	0.48 (0.87)	0.580	1.61 [0.29–8.90]		89	22
1-2 cigarettes per day	0.47 (0.92)	0.612	1.60 [0.26–9.81]	0.032	32	7
3-4 cigarettes per day	20.28 (15191.51)	0.999	6.46*∗*10^10^ [0–]		7	0
Alcohol consumption						
Never	−19.83 (40192.01)	1.000	0		97	25
Once a month	−19.64 (40192.01)	1.000	0		19	4
2-3 times a month	−19.81 (40192.01)	1.000	0	0.018	12	3
2-3 times a week	0 (49225.35)	1.000	1.00		2	0
3-4 times a week	0 (56840.76)	1.000	1.00		1	0
Drug addiction history						
Never	−0.81 (1.07)	0.445	0.44 [0.05–3.61]		123	31
In past but not currently	19.00 (40192.96)	1.000	1.79*∗*10^10^ [0–]	0.011	1	0
HIV diagnosed						
Less than a year ago	19.79 (40192.96)	1.000	3.94*∗*10^10^ [0–]	0.005	1	0
1–3 years ago	0.19 (1.11)	0.858	1.22 [0.13–10.82]		5	1
Start of ART						
Less than a year ago	−0.32 (1.17)	0.779	0.72 [0.07–7.16]	0.001	3	1
1–3 years ago	0.18 (1.11)	0.870	1.20 [0.13–10.65]		5	1
Disclosure of HIV status						
No	0.38 (1.09)	0.728	1.46 [0.17–12.612]	0.001	6	1
Educational intervention received						
No	−1.50 (0.84)	0.075	0.22 [0.04–1.16]	0.028	3	3

**Table 7:** Binary logistic regression analysis of respondents' attitude towards antiretroviral medicines with various predictor variables.

Predictors	*B* (SE)	*p* value	95% CI for OR [lower–upper]	Nagelkerke R2	Attitude	
					Positive (*n*)	Negative (*n*)
Age (years)						
18–38	1.19 (0.55)	0.031	3.29 [1.11–9.71]	0.04	41	27
39–58	0.97 (0.54)	0.072	2.66 [0.91–7.72]		42	34
Gender						
Male	0.11 (0.31)	0.729	1.11 [0.59–2.08]	0.001	54	43
Education level						
Illiterate	0.41 (0.67)	0.540	1.51 [0.40–5.73]		24	19
Just read and write	0.18 (0.64)	0.779	1.20 [0.33–4.27]	0.021	37	37
Secondary level	0.79 (0.69)	0.254	2.21 [0.56–8.67]		24	13
Occupation						
Service holders	0 (32226.15)	1.000	1.00 [0–]		2	0
Businessman	−21.29 (15191.68)	0.999	0 [0–]		11	12
Labor	−20.69 (15191.68)	0.999	0 [0–]	0.129	15	9
Agriculture	−21.30 (15191.68)	0.999	0 [0–]		47	52
Unemployed	−19.81 (15191.68)	0.999	0 [0–]		8	2
Smoking habit						
Never smoked	0.48 (0.78)	0.537	1.62 [0.34–7.61]		61	49
1-2 cigarettes per day	0.28 (0.82)	0.728	1.33 [0.26–6.73]	0.031	20	19
3-4 cigarettes per day	2.07 (1.32)	0.116	8.00 [0.59–106.93]		6	1
Alcohol consumption						
Never	21.44 (40189.82)	1.000	2.06*∗*10^11^ [0–]		69	54
Once a month	20.94 (40189.82)	1.000	1.24*∗*10^11^ [0–]		10	13
2-3 times a month	21.33 (40189.82)	1.000	1.84*∗*10^11^ [0–]	0.051	8	7
2-3 times a week	42.40 (49223.56)	0.999	2.60*∗*10^18^ [0–]		2	0
3-4 times a week	42.40 (56839.21)	0.999	2.60*∗*10^18^ [0–]		1	0
Drug addiction history						
Never	1.08 (0.70)	0.127	2.95 [0.73–11.84]	0.03	86	68
In past but not currently	22.05 (40192.96)	1.000	3.76*∗*10^11^ [0–]		1	0
HIV diagnosed						
Less than a year ago	21.00 (40192.96)	1.000	1.31*∗*10^11^ [0–]	0.019	1	0
1–3 years ago	−0.89 (0.88)	0.309	0.40 [0.07–2.29]		2	4
Start of ART						
Less than a year ago	0.93 (1.16)	0.425	2.53 [0.25–24.91]	0.006	3	1
1–3 years ago	−0.16 (0.83)	0.840	0.84 [0.16–4.31]		3	3
Disclosure of HIV status						
No	21.10 (15191.51)	0.999	1.45*∗*10^11^ [0–]	0.069	7	0
Educational intervention received						
No	−0.90 (0.88)	0.303	0.40 [0.07–2.26]	0.009	2	4

**Table 8:** Association of respondents' attitude towards antiretroviral medications with their corresponding knowledge level.

Category of knowledge of ART	Attitude category		Total	*χ* ^2^ value	*p* value
	Negative	Positive			
Poor	34 (64.2)	19 (35.8)	53 (100)	11.008 (d*f* = 1)	0.001
Good	41 (36.6)	71 (63.4)	112 (100)		
Total	75 (45.5)	90 (54.5)	165 (100)		

**Table 9:** Adherence of PLHIV towards antiretroviral therapy (*n* = 165).

Variables	Frequency (%)	
	Yes	No
(1) Have you ever missed a dose of your ART drugs?	6 (3.6)	159 (6.4)
1(a) What is the probable reason for missing a dose of ART medicine?		
Forget to take medicine	62 (37.6)	103 (62.4)
Lack of information	4 (2.4)	161 (97.6)
Difficult to swallow the drug	19 (11.5)	146 (88.5)
To avoid side effects	7 (4.2)	158 (95.8)
No access to medication	7 (4.2)	158 (95.8)
More than one tablet is to be taken once	1 (0.6)	164 (99.4)
Felt better and hence did not take pills	58 (35.2)	107 (64.8)
No any improvement seen on medications	6 (3.6)	159 (96.4)
Not wanting other people to notice	26 (15.8)	139 (84.2)
Drug regimen is difficult to follow	4 (2.4)	161 (97.6)
Too busy in other work	102 (61.8)	63 (38.2)
Away from home	85 (51.5)	80 (48.5)
Felt depressed	51 (30.9)	114 (69.1)
Felt too ill	36 (21.8)	129 (78.2)
Ran out of pills	23 (13.9)	142 (86.1)
Physical disability	29 (17.6)	136 (82.4)
Others	2 (1.2)	163 (98.8)
(2) How do you remember to take your ART?		
No particular method	114 (69.1)	51 (30.9)
Help from a family/friend/relative	46 (27.9)	119 (72.1)
Reminder device	—	165 (100)
Others	3 (1.8)	162 (98.2)
(3) Have you faced any side effects of the ART medications that you are taking?	2 (1.2)	163 (98.8)

**Table 10:** Binary logistic regression analysis of patients' adherence to antiretroviral medicines with various predictor variables.

Predictors	*B* (SE)	*p* value	95% CI for OR [lower–upper]	Nagelkerke R2
Age (years)				
18–38	−17.70 (9220.89)	0.998	0	0.03
39–58	−17.56 (9220.89)	0.998	0	
Gender				
Male	−0.76 (1.16)	0.514	0.46 [0.04–4.59]	0.014
Education level				
Illiterate	0 (13580.53)	1.000	1.000 [0–]	
Just read and write	−17.61 (12118.65)	0.999	0 [0–]	0.109
Secondary level	−18.34 (12118.65)	0.999	0 [0–]	
Occupation				
Service holders	−21.20 (15191.58)	0.999	0 [0–]	
Businessman	0 (17349.99)	1.000	1.00 [0–]	
Labor	−18.06 (15191.58)	0.999	0 [0–]	
Agriculture	0 (15719.48)	1.000	1.00 [0–]	
Unemployed	−19.81 (15191.58)	0.999	0 [0–]	
Smoking habit				
Never smoked	2.20 (1.29)	0.088	9.08 [0.71–114.85]	
1-2 cigarettes per day	1.87 (1.48)	0.206	6.50 [0.35–118.37]	0.075
3-4 cigarettes per day	19.41 (15191.51)	0.999	2.69*∗*10^10^ [0–]	
Alcohol consumption				
Never	−17.51 (40192.97)	1.000	0 [0–]	
Once a month	0 (41057.44)	1.000	1.00 [0–]	
2-3 times a month	0 (41511.12)	1.000	1.00 [0–]	0.273
2-3 times a week	0 (49226.13)	1.000	1.00 [0–]	
3-4 times a week	−42.40 (56841.44)	0.999	0 [0–]	
Drug addiction history				
Never	−17.57 (12710.13)	0.999	0 [0–]	0.017
In past but not currently	0 (42154.74)	1.000	1.00 [0–]	
HIV diagnosed				
Less than a year ago	17.25 (40192.96)	1.000	3.12*∗*10^9^ [0–]	0.074
1–3 years ago	−2.33 (1.24)	0.060	0.09 [0.009–1.10]	
Start of ART				
Less than a year ago	17.27 (20096.48)	0.999	3.18*∗*10^9^ [0–]	0.078
1–3 years ago	−2.31 (1.24)	0.062	0.09 [0.009–1.12]	
Disclosure of HIV status				
No	17.55 (15191.51)	0.999	4.19*∗*10^9^ [0–]	0.01
Educational intervention received				
No	17.54 (16408.71)	0.999	4.16*∗*10^9^ [0–]	0.009

## Data Availability

The readers can access the data from the supplementary files and the additional data can be made available to readers on request to the corresponding author.

## Abbreviations

ART: Antiretroviral therapy
ADC: ART dispensing centers
CHBC: Community and home-based care
CVR: Content validity ratio
C-CVI: Clarity content validity index
DTG: Dolutegravir
EFV: Efavirenz
INSTI: Integrase strand transfer inhibitor
MoHP: Ministry of Health and Population
MTCT: Mother-to-child HIV transmission
NHRC: Nepal Health Research Council
NRTIs: Nucleoside reverse transcriptase inhibitors
PDR: Lao People's Democratic Republic
PLHIV: People living with HIV
R-CVI: Relevance content validity index
S-CVI: Simplicity content validity index
SPSS: Statistical Package for Social Sciences
TLD: Tenofovir, lamivudine, and dolutegravir
UNAIDS: Joint United Nations Programme on HIV/AIDS
WHO: World Health Organization.

## Supplementary Materials

Supplementary Materials Supplementary file: questionnaire.
