# Supplementary material for: Knowledge, Attitude, Practice, and Adherence to Antiretroviral Therapy among People Living with HIV in Nepal
Source: AIDS Res Treat. 2023 Jul 17;2023:7292115. doi: 10.1155/2023/7292115 (PMC10365915; doi:10.1155/2023/7292115)
Supplement: Supplementary Materials — Supplementary file: questionnaire. [file 7292115.f1.docx]

**Quantative Study Questionnaire**

**Proposed data collection sheet**

**Patient No.: …………… ART Site:……………**

1. **(1) SOCIODEMOGRAPHIC INFORMATION**
2. Age: ………………….. Years Old
3. Gender:
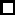
 Male
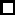
 Female
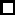
 Transgender
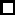
 Others (Please Specify): ………………….
4. Religion:
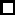
 Hindu
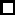
Buddhist
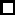
 Muslim
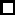
Christian


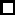
Others (Please Specify): ……

1. Marital Status:
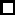
Married
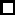
Unmarried
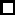
Divorced
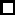
 Single


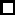
 Others (Please Specify): ………………….

1. Education:
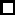
 Can’t read and write
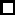
 Just read and write
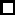
Secondary


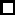
Bachelor
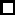
Bachelor and above

1. Occupation:
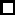
Service
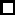
Business
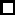
 Labor
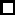
 Agriculture


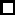
 Unemployed
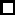
 Others (Please Specify): ………………….

1. Monthly income (NRS):
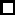
Less than 10,000
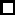
10,000-20,000
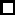
 More than 20,000
2. **(2) SOCIODEMOGRAPHIC INFORMATION**
3. Smoking in past 1 month:


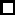
Never
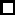
1-2 cigarettes per day
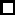
3-4 cigarettes per day
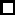
More than 4 cigarettes per day

1. Alcohol use in past 1 month:
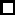
 Never
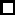
 Once a month
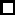
 2-3 times a month
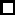
 2-3 times a week
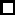
 3-4 times a week
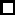
 Daily
2. Were you ever a drug addict?


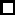
 Never
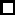
 In past but not currently
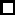
 Yes, currently

1. You were diagnosed with HIV:
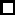
Less than a year ago
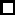
 1- 3 years ago
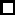
More than 3 years ago
2. You started ART:
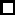
Less than a year ago
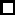
 1- 3 years ago
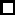
More than 3 years ago
3. Disclosure of HIHhhhhHMJJMFDHVJM HIV status:
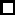
 Yes
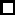
No

If **Yes**, to whom?


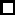
Wife
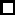
Mother
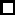
Father
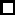
Brother
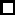
Children’s
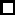
 Friends
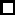
Sexual Partner

1. Time to reach ART site:
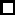
 Less than or equal to 1 hour
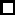
 More than 1 hour
2. Have you received any educational intervention regarding HIV or ART?
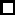
Yes
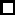
No

If **Yes**, from whom?


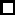
Physician
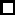
 Pharmacy staff providing HIV drugs
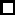
Institution of HIV
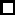
 Psychological counselor
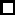
Others (Please Specify)……………….

1. **KNOWLEDGE ABOUT HIV/AIDS**
2. Is HIV a hereditary disease?
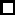
Yes 
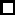
 No
3. Are HIV and AIDS the same thing?
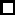
Yes 
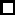
 No
4. So far, HIV vaccine has been developed yet?
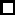
True 
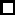
 False

If not, then scientists are working to make one?
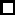
True 
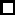
 False

1. Which one of the following is not the mode of transmission of HIV?

- Unsafe sex
- Syringes
- Mother to child transmission
- Blood transfusion
- Mosquito bite

1. Does breastfeeding transmit HIV?
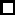
Yes 
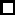
 No
2. Window period is

- When HIV cannot be seen in the blood
- When HIV can be seen in the blood
- When HIV cannot be seen in the lymph node
- When HIV can be seen in the lymph node
- Don’t Know.

1. HIV viral load is
   - Number of fighter cells in the blood
   - Number of copies of HIV in blood
2. If you are HIV positive, your children may be positive?
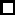
 Yes 
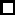
 No
3. **KNOWLEDGE OF ANTIRETROVIRAL THERAPY**
4. What is the name of your ART medication?
5. TDF+3TC+DTG Correct/Incorrect 1tab OD
6. ABC+3TC+DTG Correct/Incorrect (ABC+3TC=1tab and DTG=1tab OD)
7. AZT+3TC+DTG Correct/Incorrect (AZT+3TC=1tab BD and DTG=1tab OD)
8. AZT+3TC+ATV/r (or LPV/r)Correct/Incorrect (AZT+3TC=1tab BD and LPV/r 200/50mg=2tab BD)

Note: TDF=Tenofovir disoproxil, 3TC=Lamivudine. DTG=Dolutegravir, ABC=Abacavir, AZT=Zidovudine, EFV=Efavirenz, LPV/r=Lopinavir/ritonavir, ATV=Atazanavir

1. How many tablets should you take each day for your ART medication?
2. One tab Correct/Incorrect
3. Two tab Correct/Incorrect
4. Three tab Correct/Incorrect
5. Four tab Correct/Incorrect
6. Five tab Correct/Incorrect
7. Six tab Correct/Incorrect
8. How should you take your ART?
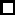
At a fixed time
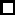
At a variable time
9. How should you take your ART in relation to food intake?


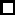
After food
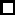
 Half an hour before meal
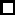
Either with or without food

1. How long should you take your ART?
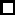
 Lifelong
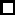
For some years


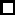
 Can be stopped by doctor depending on CD4 count
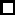
 Don’t Know

1. What is the main purpose of ART?

- Suppress the power of HIV
- It does not cure the disease forever
- It suppress the power of HIV but does not cure the disease forever
- Cure HIV/AIDS
- Don’t Know

1. What is the effect of ART on HIV viral load?


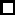
Decreases HIV viral load
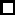
 Increases HIV viral load
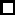
 Does nothing to HIV viral load
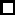
Don’t Know

1. What is the effect of ART on CD4 count?

- Decreases CD4 cells count
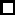
Increases CD4 cells count
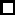
Don’t Know

1. What is the effect of ART on mother to child HIV transmission?

- It can prevent transmission
- It can’t prevent transmission
- Don’t know

1. How much percentage of ART adherence is required to achieve optimum supression of HIV viral load?
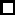
Upto 94%
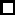
 95-100%
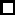
Don’t Know
2. Does missing your ART medication reduce treatment efficiency?
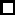
 Yes
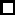
 No
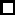
Don’t Know
3. **ATTITUDE TOWARDS ANTIRETROVIRAL THERAPY**
4. Believe to have effective therapy to treat HIV other than ART

| - Strongly Disagree | - Disagree | - Undecided | - Agree | - Strongly Agree |
| --- | --- | --- | --- | --- |

1. I needn't bother with ARV drugs since I'm not persuaded that I have HIV/AIDS

| - Strongly Disagree | - Disagree | - Undecided | - Agree | - Strongly Agree |
| --- | --- | --- | --- | --- |

1. ART has positive effect on health

| - Strongly Disagree | - Disagree | - Undecided | - Agree | - Strongly Agree |
| --- | --- | --- | --- | --- |

1. ART gives more harm than benefits.

| - Strongly Disagree | - Disagree | - Undecided | - Agree | - Strongly Agree |
| --- | --- | --- | --- | --- |

1. Taking ARV drugs for one’s lifetime is tiring.

| - Strongly Disagree | - Disagree | - Undecided | - Agree | - Strongly Agree |
| --- | --- | --- | --- | --- |

1. It is shameful to be on ARV therapy.

| - Strongly Disagree | - Disagree | - Undecided | - Agree | - Strongly Agree |
| --- | --- | --- | --- | --- |

1. ARV drugs help to prolong life.

| - Strongly Disagree | - Disagree | - Undecided | - Agree | - Strongly Agree |
| --- | --- | --- | --- | --- |

1. You should take ART only when you feel sick.

| - Strongly Disagree | - Disagree | - Undecided | - Agree | - Strongly Agree |
| --- | --- | --- | --- | --- |

1. **PRACTICE REGARDING ANTIRETROVIRAL THERAPY**
2. Where do you store your ART at home?
   - Hidden and out of sight
   - Convenient storage but not necessarily as recommended by thedispenser
   - Storage that can help to remember daily schedule but not necessarily as recommend by dispenser
   - Suitable storage as recommended by the manufacturer
   - Storage out of the reach and sight of children
3. **How do you store your ART at home?**

- By transferring to other plastic packaging
- In its original carton or plastic packaging or bottle

1. You just remembered that you forgot to take your evening ART medication dose yesterday. You would:

- Skip the dose of ART medication you missed
- Take the missed ART medication dose right now only if it is not too close to the time of next dose
- Wait and take 2 doses of ART medication this evening
- Not missed

1. If you ran out of your prescription for your ART medication you would
   - Borrow from friends
   - Call and ask for refills
   - Wait until your next appointment to get a new prescription
   - Others……………………………………. specify
2. Where do you find out information about your ART? (Multiple Response Possible)

- Referring Physician
- Internet
- Patient Information Leaflet
- ART dispensing staff
- Other patients living with HIV
- HIV/AIDS organizations
- Others (Please Specify): ………………….

1. Have you ever increased or decreased the dose of your ART?
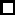
Yes
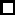
NoDon’t Know
2. Have you ever practiced self-medication? Yes NoDon’t Know

If yes, what type of drugs have you already taken in self-medication? (Multiple Response Possible)

- Antibiotics
- Antipyretic
- Drugs to relieve gastric acidity
- Painkillers
- Herbal and ayurvedicdrugs
- Others (Please Specify): ………………

1. **ADHERENCE TO ANTIRETROVIRAL THERAPY**
2. Have you ever missed a dose of your ART drugs? Yes No

If yes:

- 1. How many pills have you missed during last one month (30 days)?

……………………………………

- 1. Why did you miss a dose of your ART medicine? (Multiple Response Possible)
- Forget to take medicine
- Lack of information
- Difficult to swallow the drug
- To avoid side effects
- No access to medication
- More than one tablet to be taken once
- Felt better and hence did not take pills
- No any improvement seen on medications
- Not wanting other people to notice
- Drug regimen is difficult to follow
- Too busy in other work
- Away from home
- Felt depressed
- Felt too ill
- Ran out of pills
- Physical disability
- Others (Please Specify): ………………….

1. How do you remember to take your ART?

- No particular method
- Help from a family/friend/ relative
- Reminder device
- Others (Please Specify): ………………….

1. Have you faced any side effects of the ART medications that you are taking?Yes No

If yes what are the side effects that you have faced?

……………………………………………………………………………………

**Thank you for your time and patience!**
